# Supplementary figures and images for: Deep‐Diffeomorphic Networks for Conditional Brain Templates
Source: Hum Brain Mapp. 2025 May 15;46(8):e70229. doi: 10.1002/hbm.70229 (PMC12079767; doi:10.1002/hbm.70229)

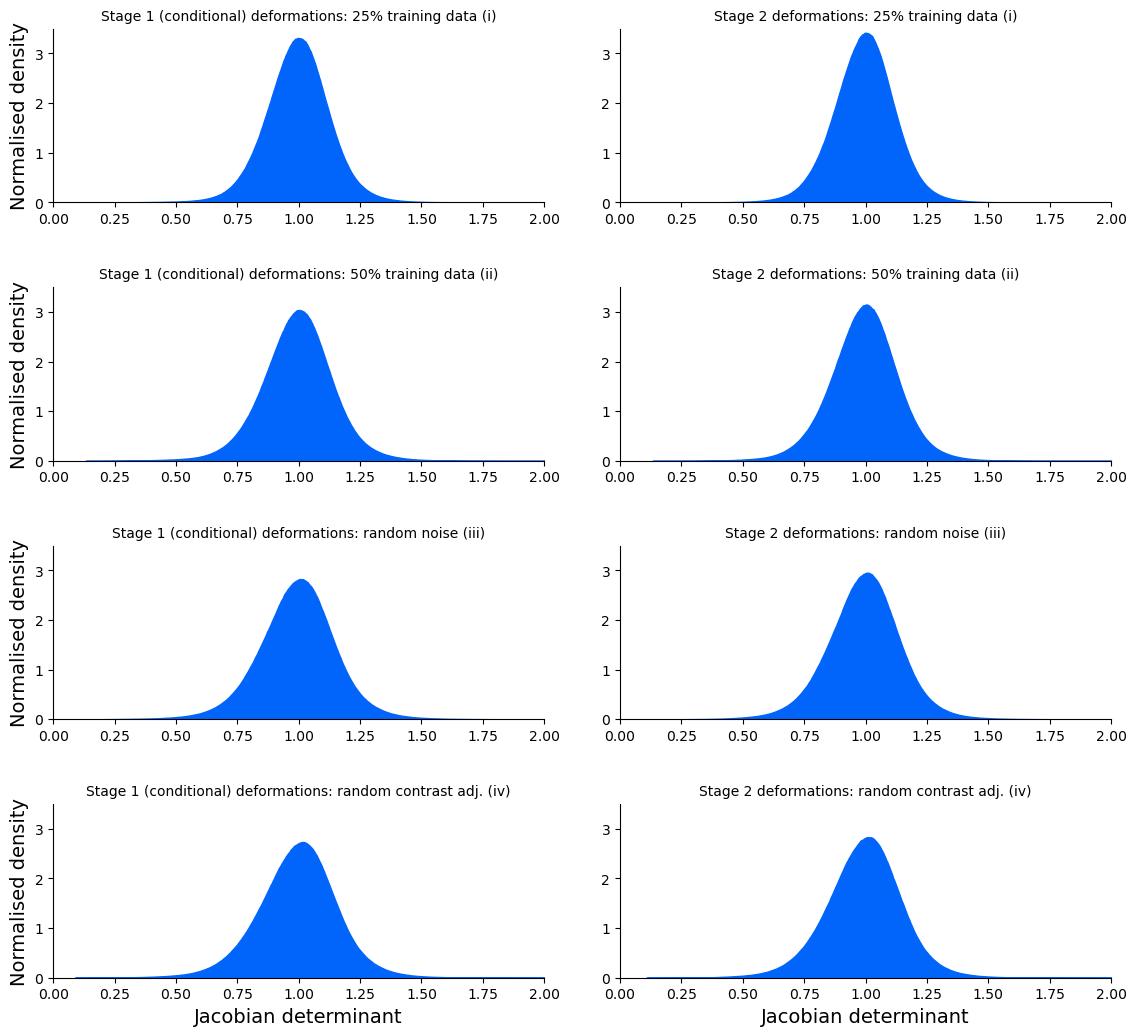

Supplement: Supplementary file 1 — Data S1. [file HBM-46-e70229-s002.zip › hbm70229-sup-0001-Supinfo/figure_1.png]

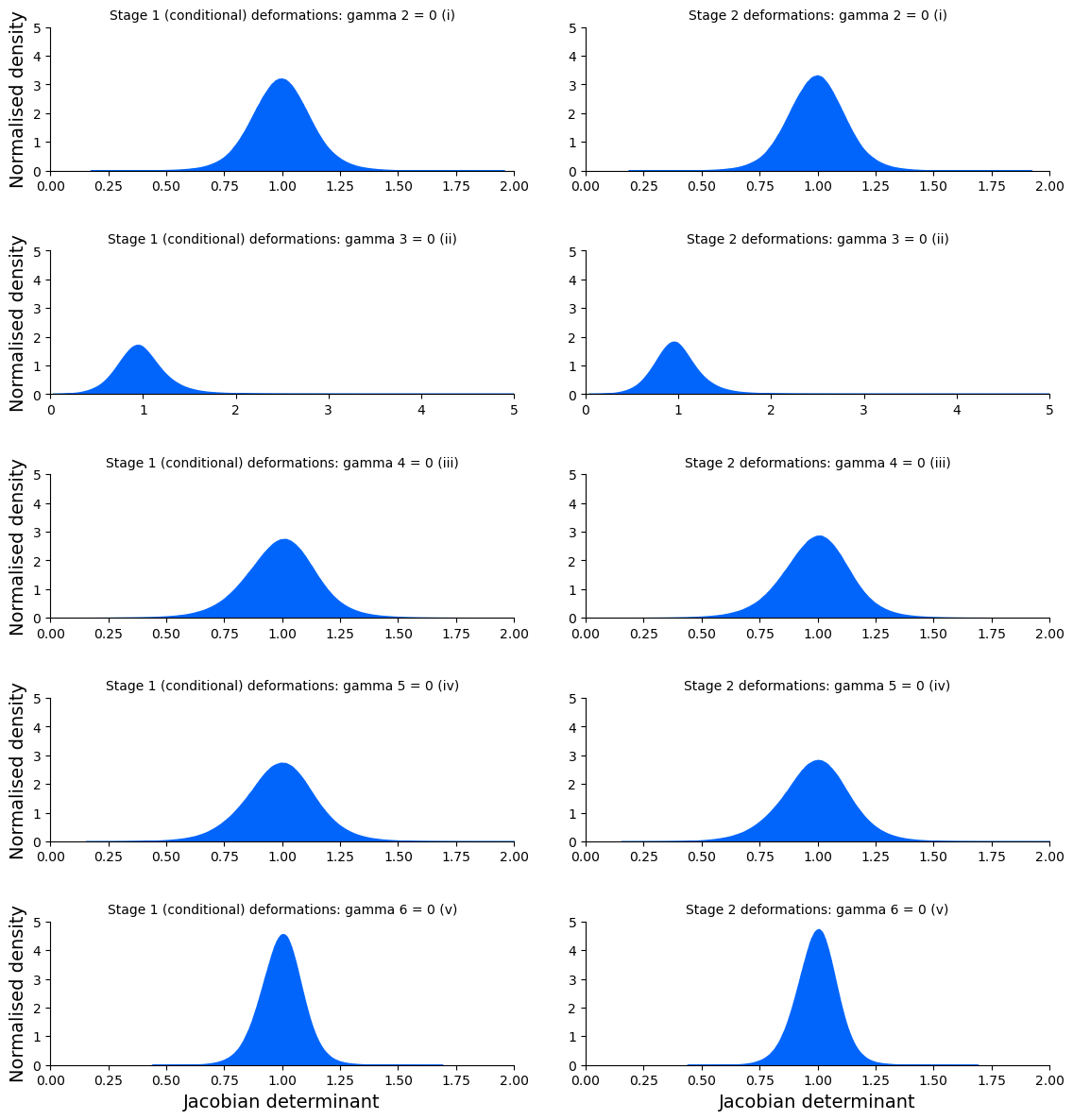

Supplement: Supplementary file 1 — Data S1. [file HBM-46-e70229-s002.zip › hbm70229-sup-0001-Supinfo/figure_10.png]

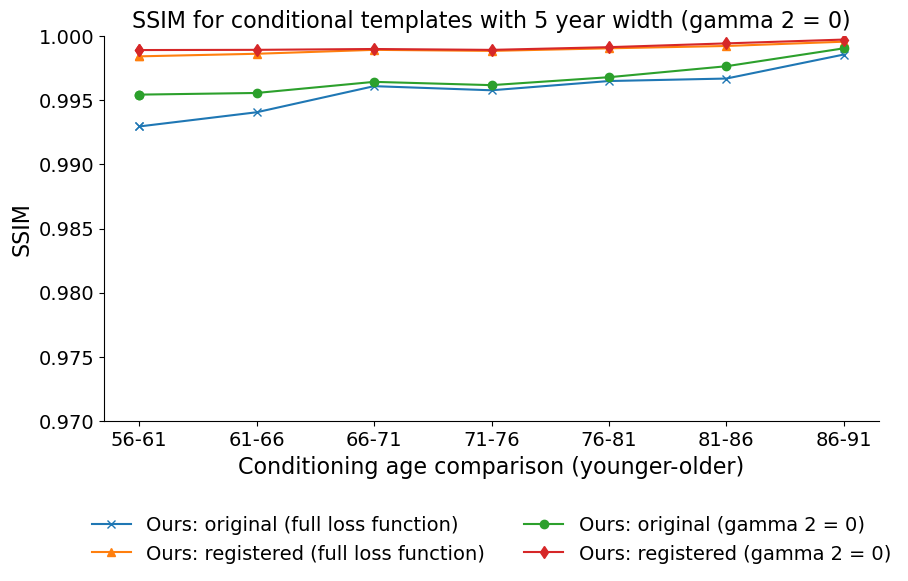

Supplement: Supplementary file 1 — Data S1. [file HBM-46-e70229-s002.zip › hbm70229-sup-0001-Supinfo/figure_11a.png]

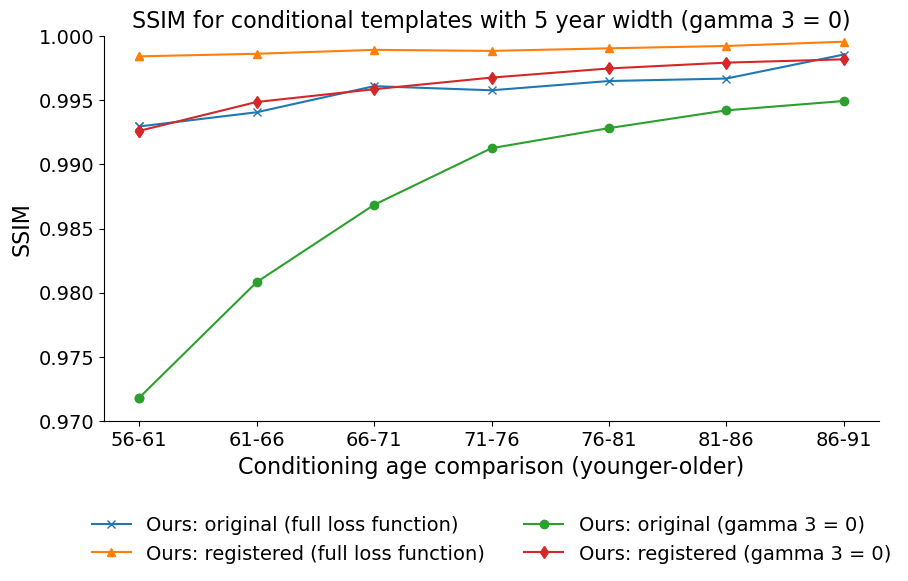

Supplement: Supplementary file 1 — Data S1. [file HBM-46-e70229-s002.zip › hbm70229-sup-0001-Supinfo/figure_11b.png]

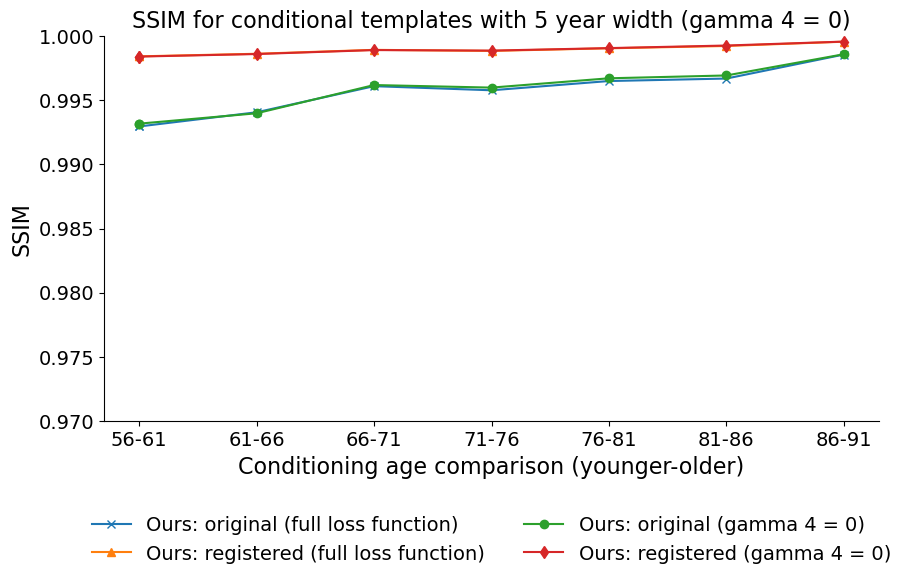

Supplement: Supplementary file 1 — Data S1. [file HBM-46-e70229-s002.zip › hbm70229-sup-0001-Supinfo/figure_11c.png]

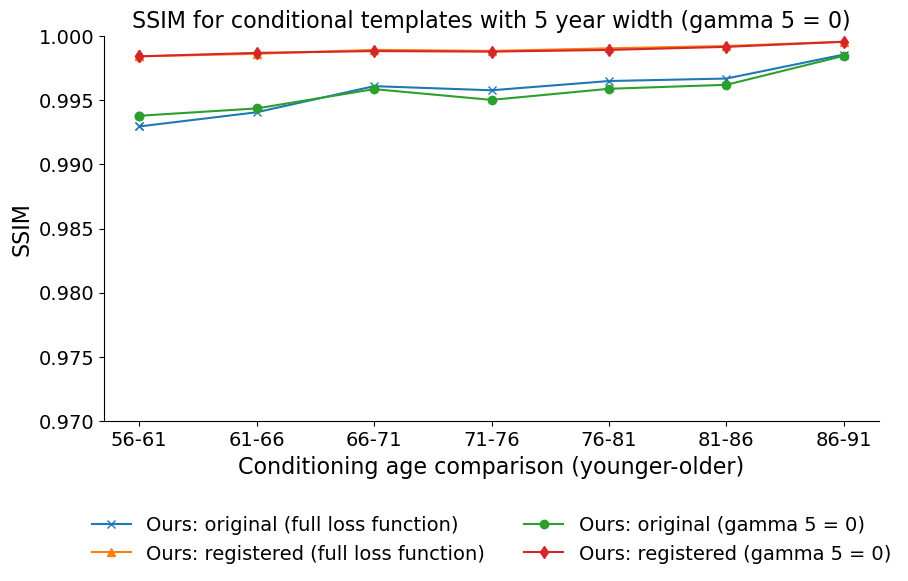

Supplement: Supplementary file 1 — Data S1. [file HBM-46-e70229-s002.zip › hbm70229-sup-0001-Supinfo/figure_11d.png]

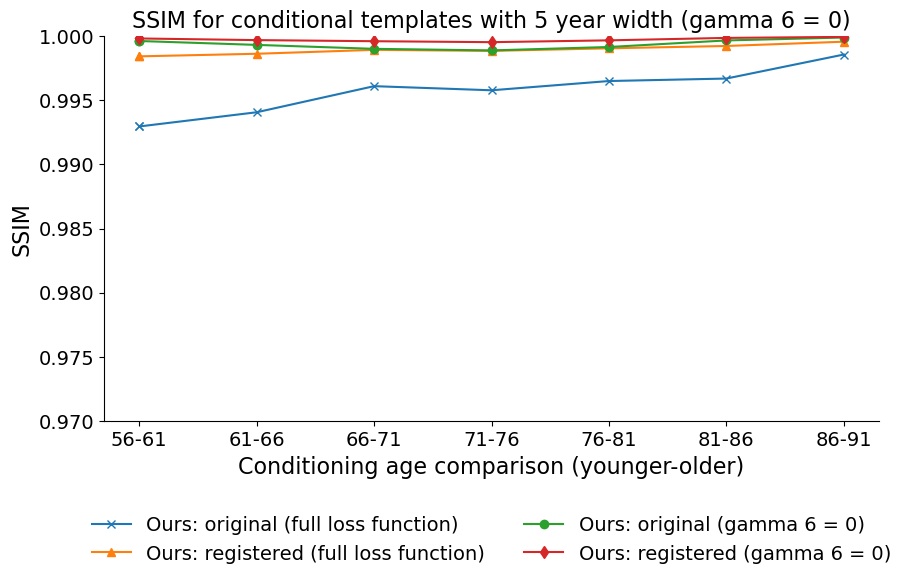

Supplement: Supplementary file 1 — Data S1. [file HBM-46-e70229-s002.zip › hbm70229-sup-0001-Supinfo/figure_11e.png]

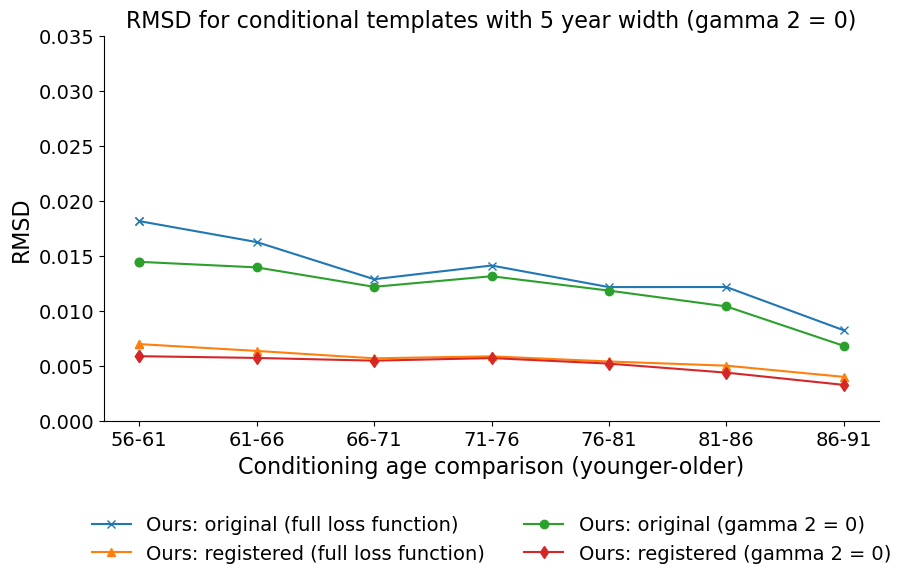

Supplement: Supplementary file 1 — Data S1. [file HBM-46-e70229-s002.zip › hbm70229-sup-0001-Supinfo/figure_12a.png]

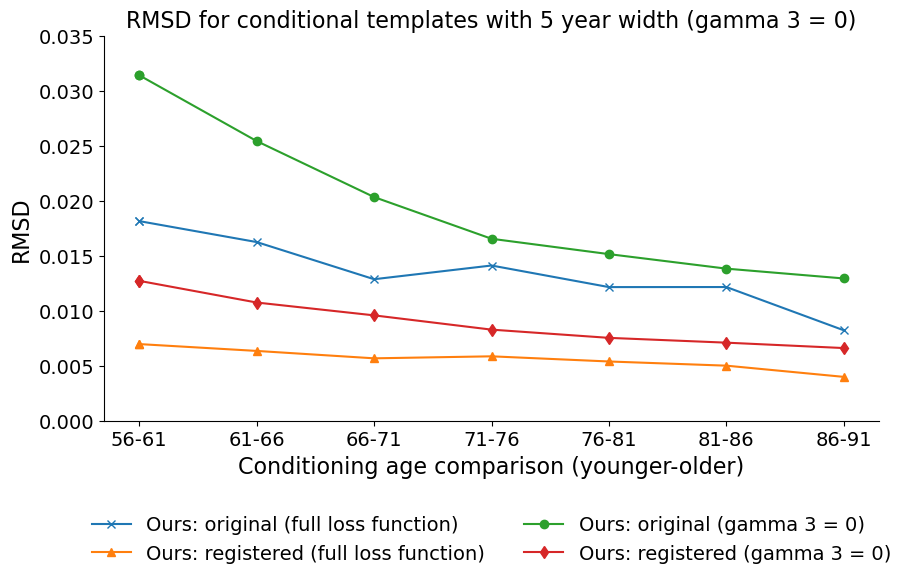

Supplement: Supplementary file 1 — Data S1. [file HBM-46-e70229-s002.zip › hbm70229-sup-0001-Supinfo/figure_12b.png]

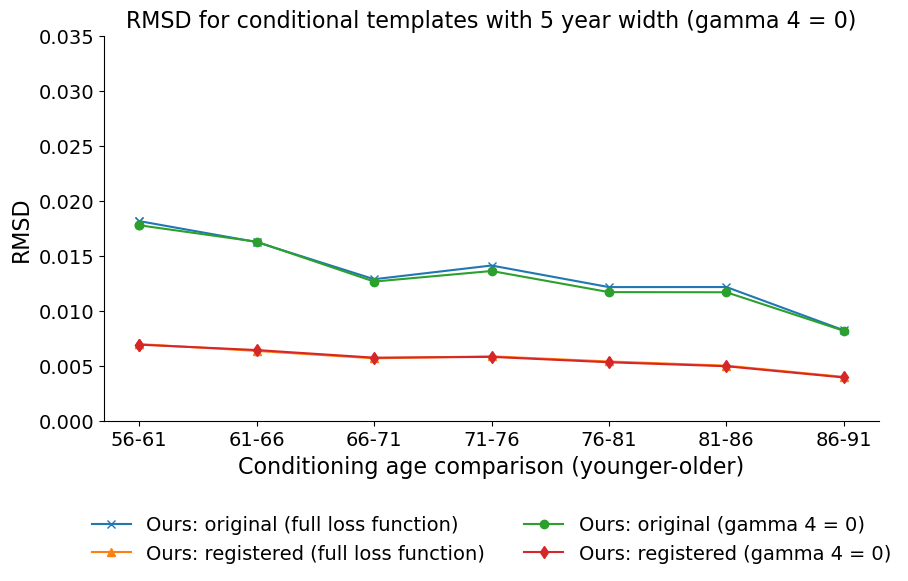

Supplement: Supplementary file 1 — Data S1. [file HBM-46-e70229-s002.zip › hbm70229-sup-0001-Supinfo/figure_12c.png]

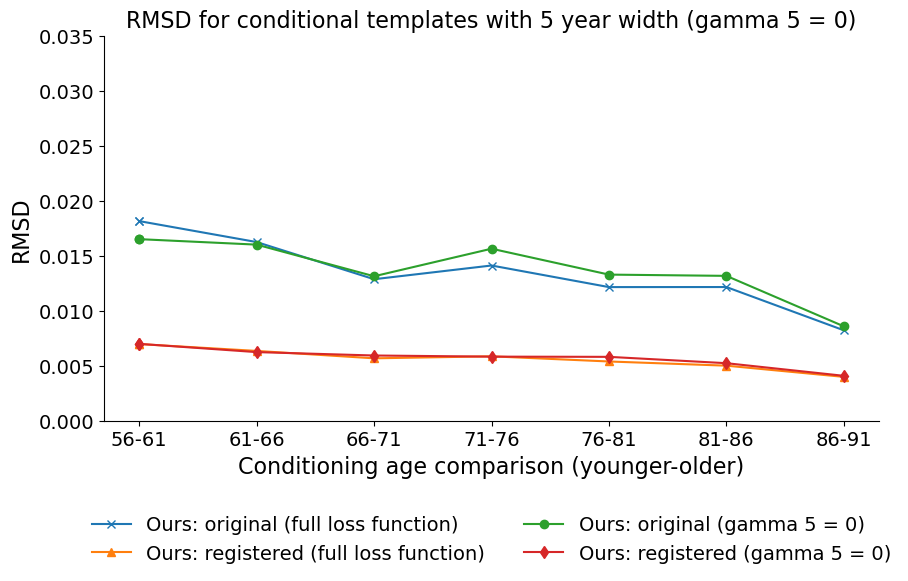

Supplement: Supplementary file 1 — Data S1. [file HBM-46-e70229-s002.zip › hbm70229-sup-0001-Supinfo/figure_12d.png]

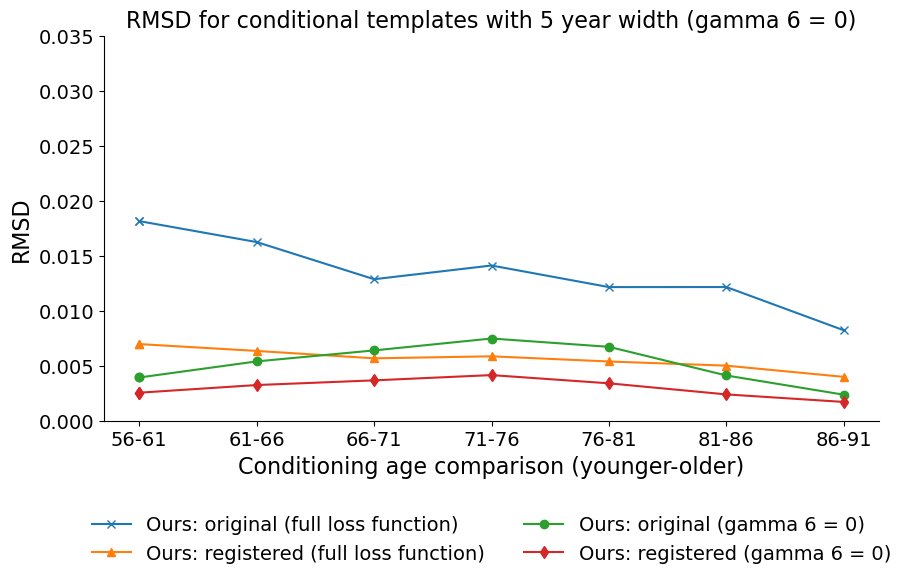

Supplement: Supplementary file 1 — Data S1. [file HBM-46-e70229-s002.zip › hbm70229-sup-0001-Supinfo/figure_12e.png]

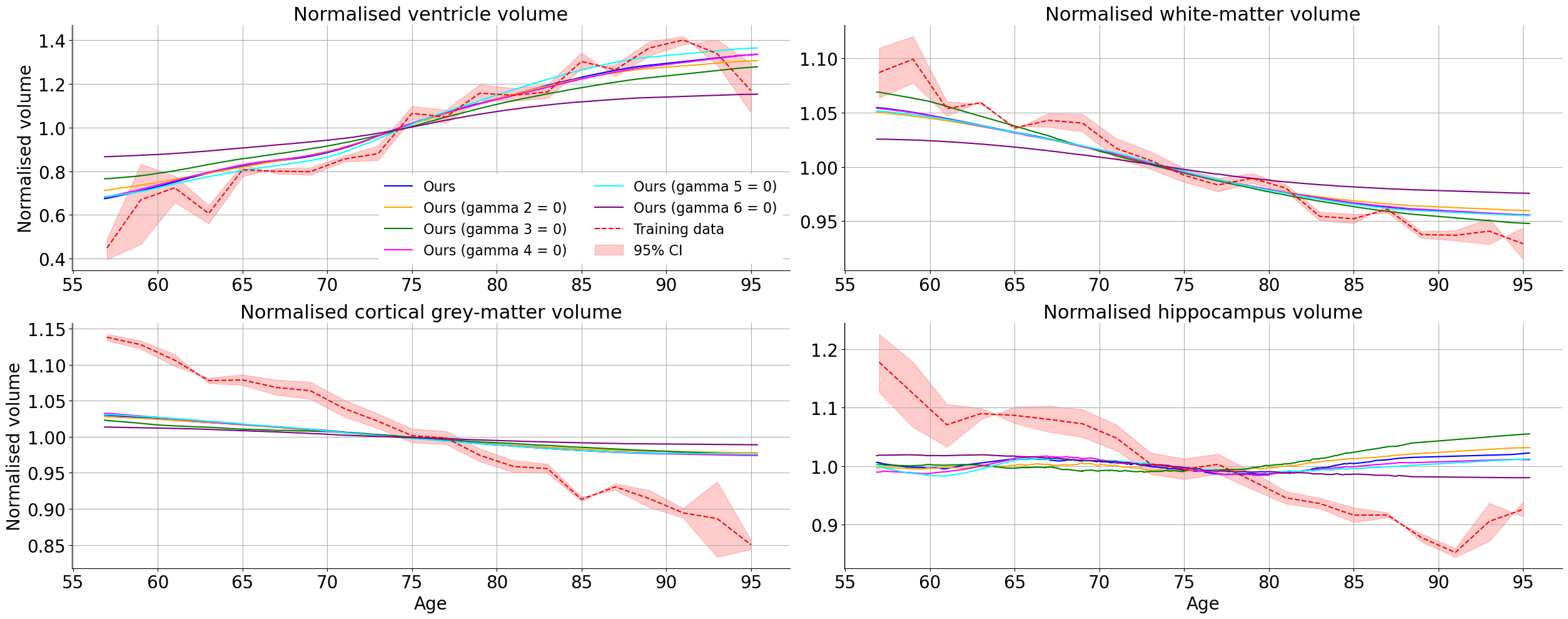

Supplement: Supplementary file 1 — Data S1. [file HBM-46-e70229-s002.zip › hbm70229-sup-0001-Supinfo/figure_13.png]

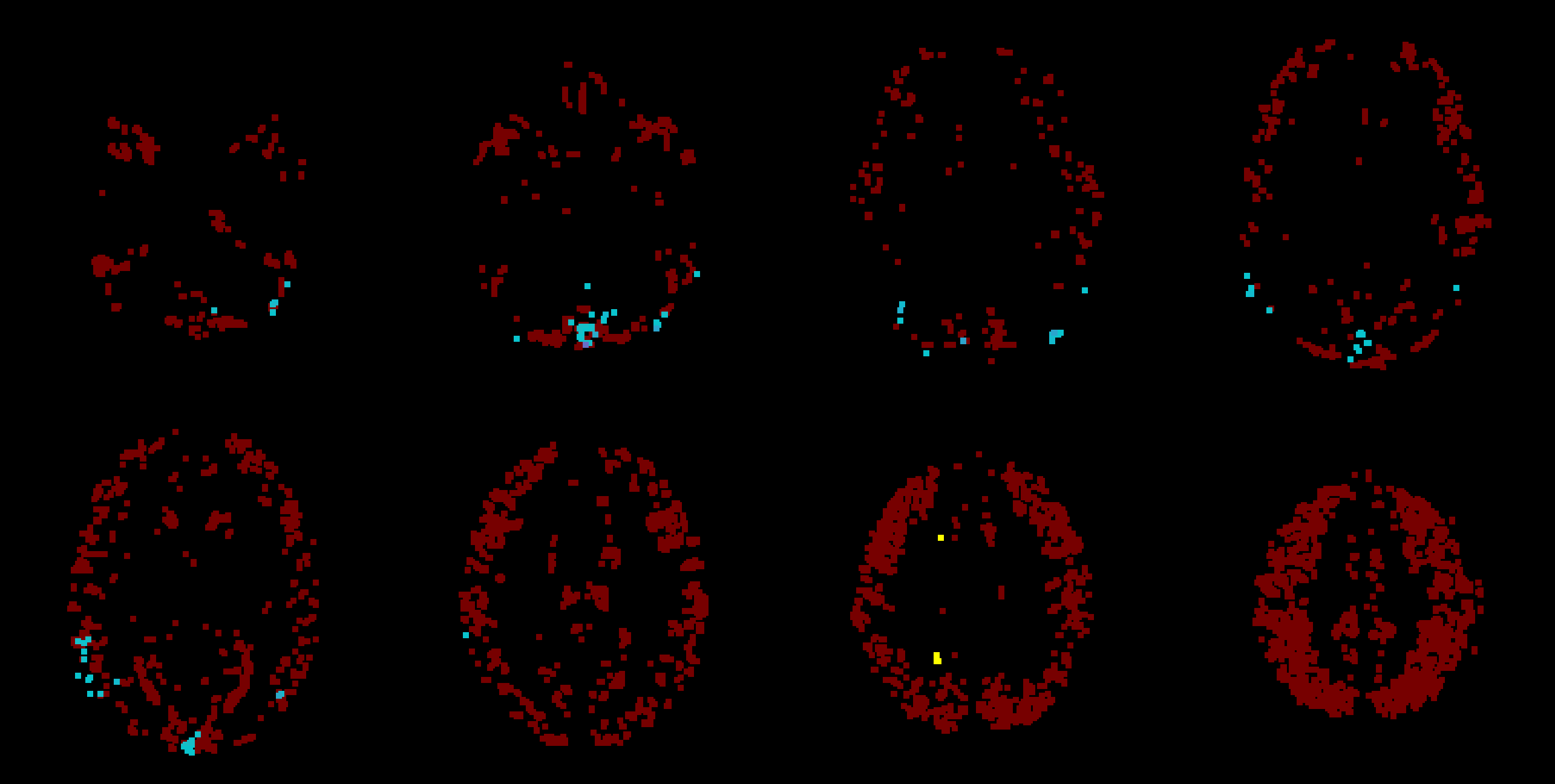

Supplement: Supplementary file 1 — Data S1. [file HBM-46-e70229-s002.zip › hbm70229-sup-0001-Supinfo/figure_14.png]

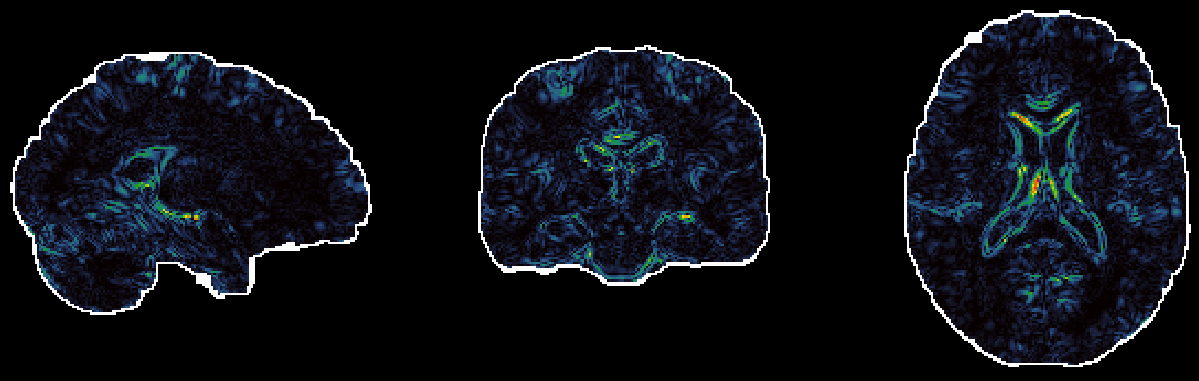

Supplement: Supplementary file 1 — Data S1. [file HBM-46-e70229-s002.zip › hbm70229-sup-0001-Supinfo/figure_15a.png]

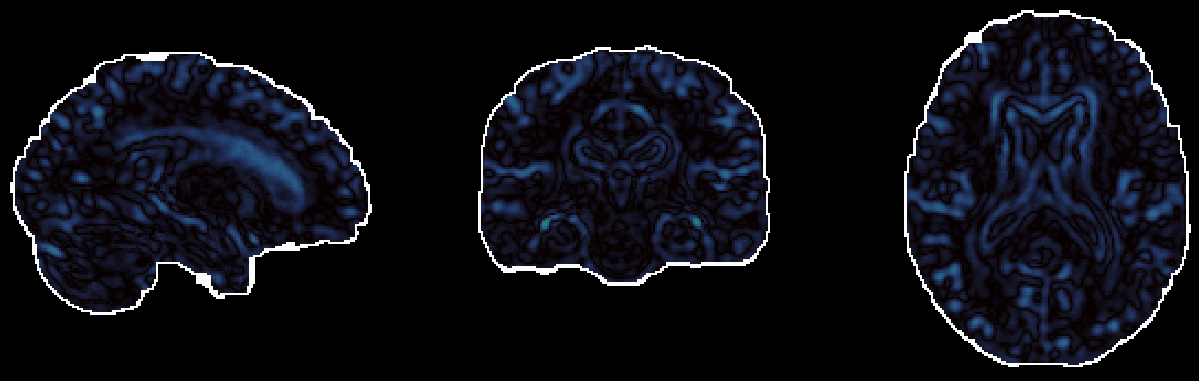

Supplement: Supplementary file 1 — Data S1. [file HBM-46-e70229-s002.zip › hbm70229-sup-0001-Supinfo/figure_15b.png]

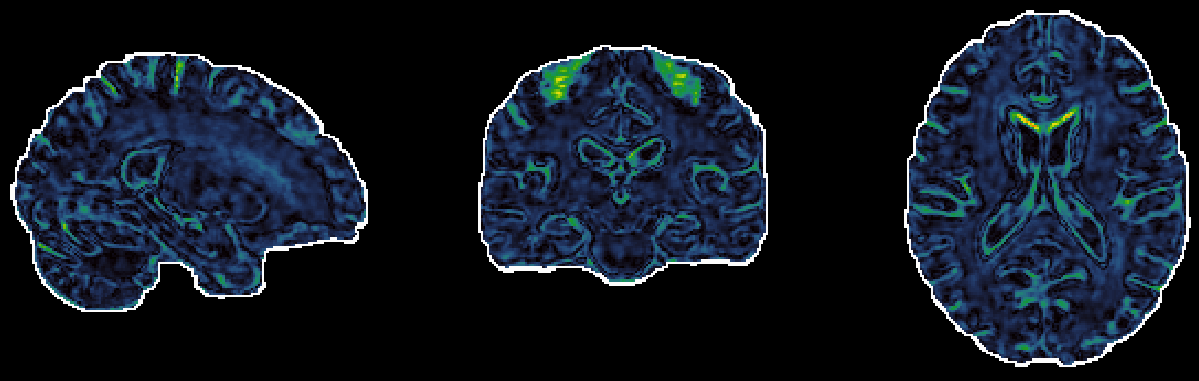

Supplement: Supplementary file 1 — Data S1. [file HBM-46-e70229-s002.zip › hbm70229-sup-0001-Supinfo/figure_15c.png]

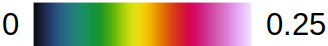

Supplement: Supplementary file 1 — Data S1. [file HBM-46-e70229-s002.zip › hbm70229-sup-0001-Supinfo/figure_15d.png]

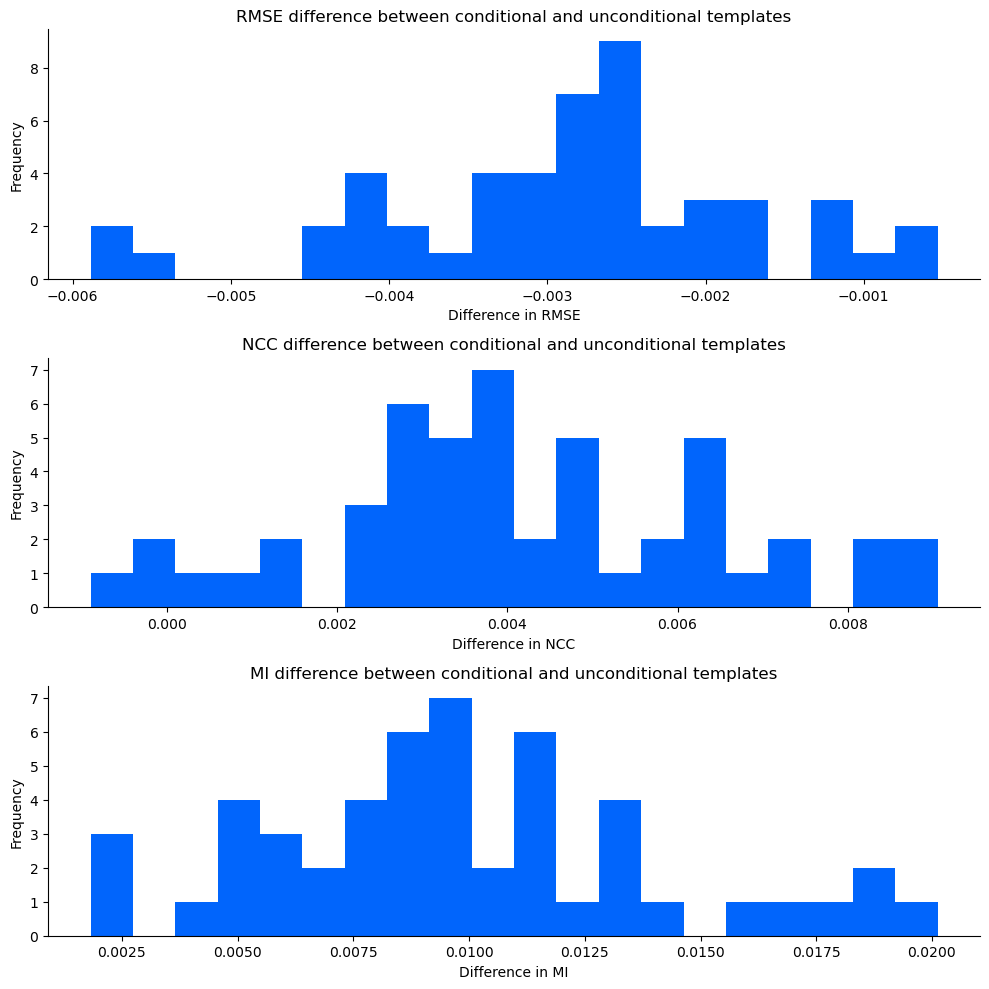

Supplement: Supplementary file 1 — Data S1. [file HBM-46-e70229-s002.zip › hbm70229-sup-0001-Supinfo/figure_16.png]

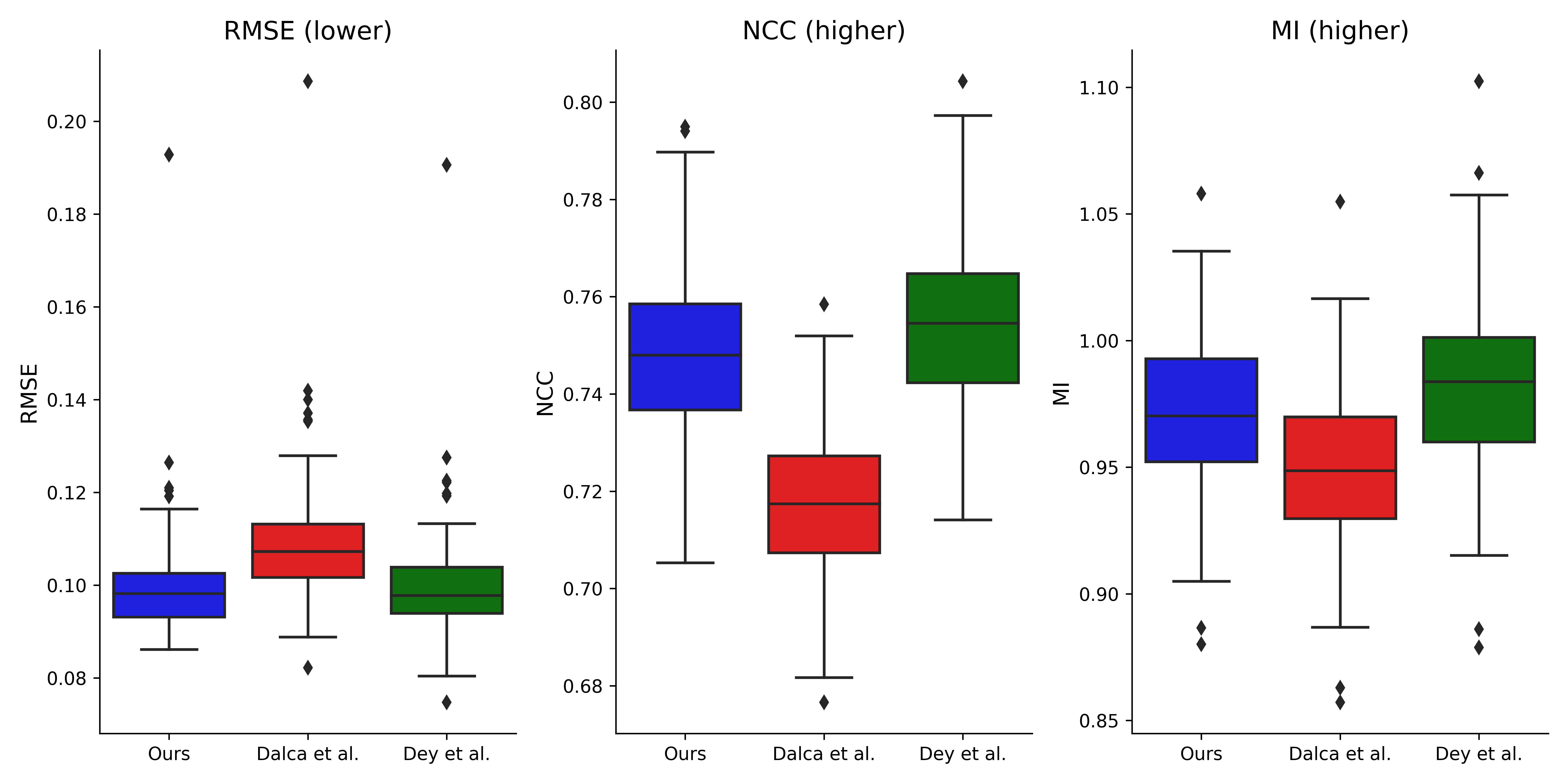

Supplement: Supplementary file 1 — Data S1. [file HBM-46-e70229-s002.zip › hbm70229-sup-0001-Supinfo/figure_17.png]

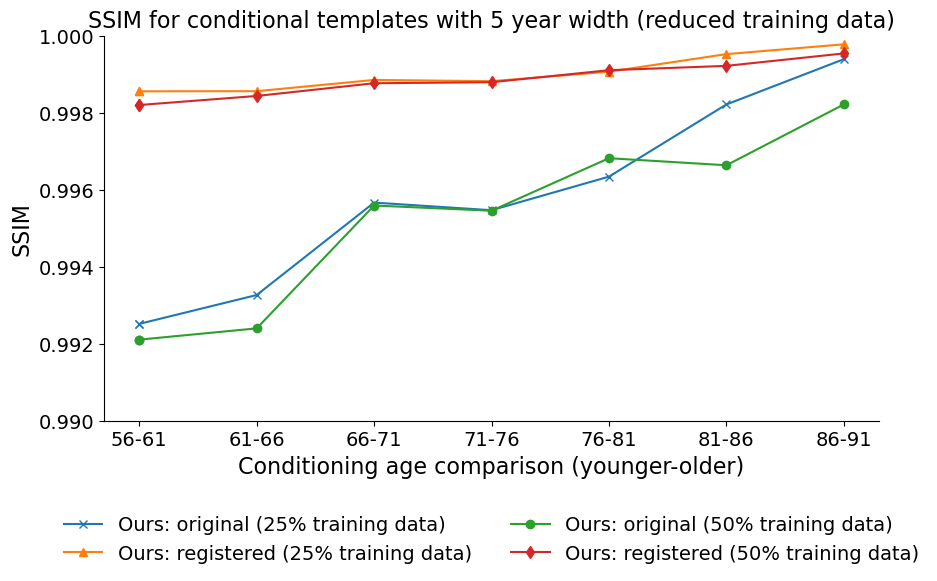

Supplement: Supplementary file 1 — Data S1. [file HBM-46-e70229-s002.zip › hbm70229-sup-0001-Supinfo/figure_2a.png]

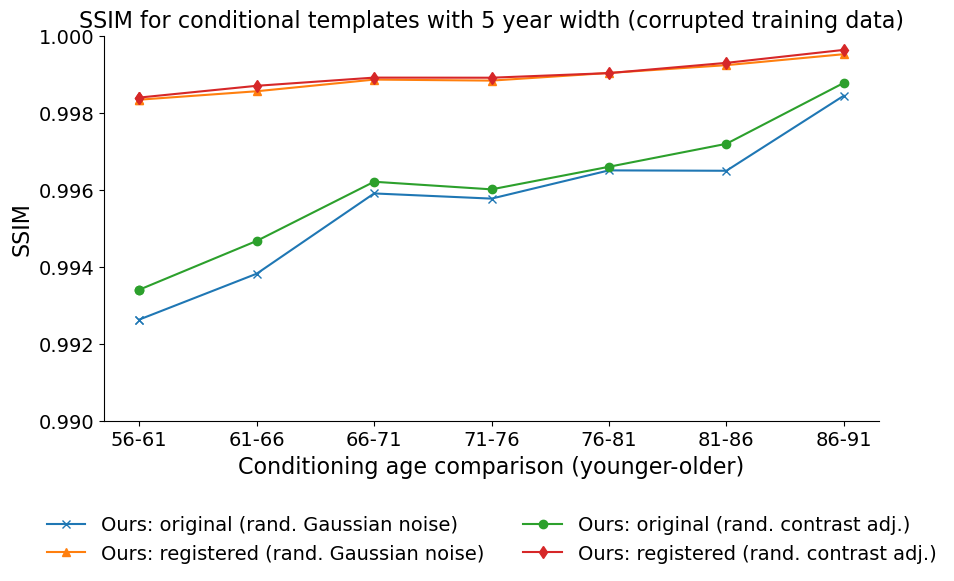

Supplement: Supplementary file 1 — Data S1. [file HBM-46-e70229-s002.zip › hbm70229-sup-0001-Supinfo/figure_2b.png]

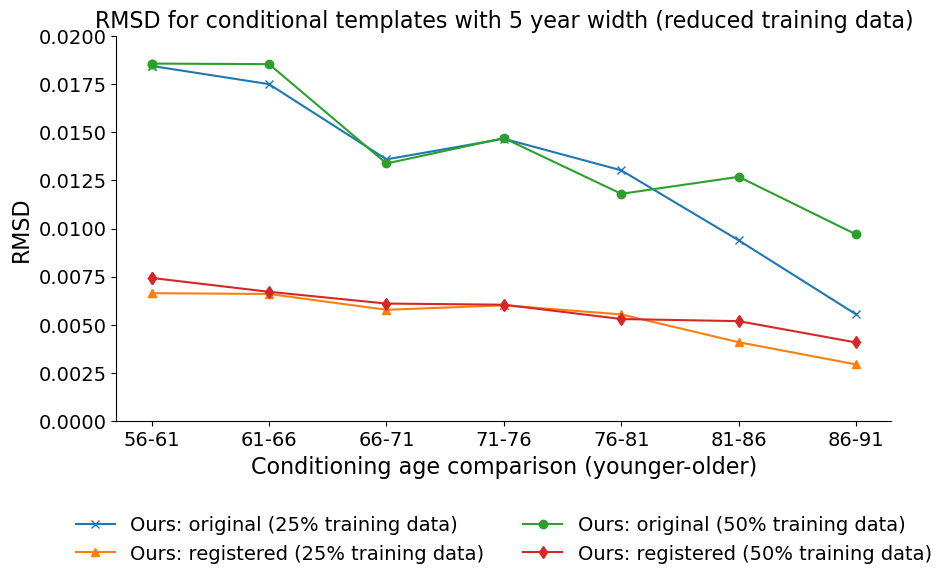

Supplement: Supplementary file 1 — Data S1. [file HBM-46-e70229-s002.zip › hbm70229-sup-0001-Supinfo/figure_2c.png]

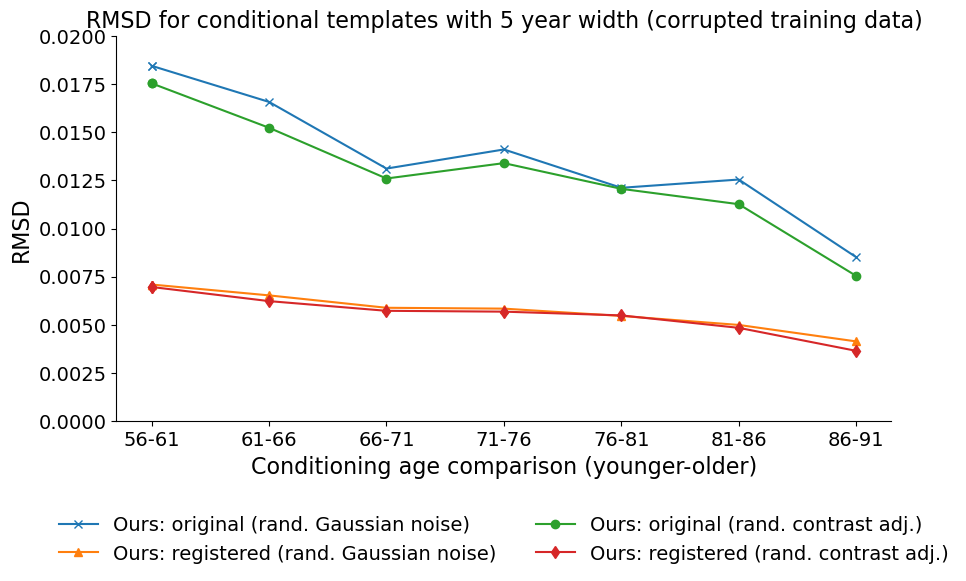

Supplement: Supplementary file 1 — Data S1. [file HBM-46-e70229-s002.zip › hbm70229-sup-0001-Supinfo/figure_2d.png]

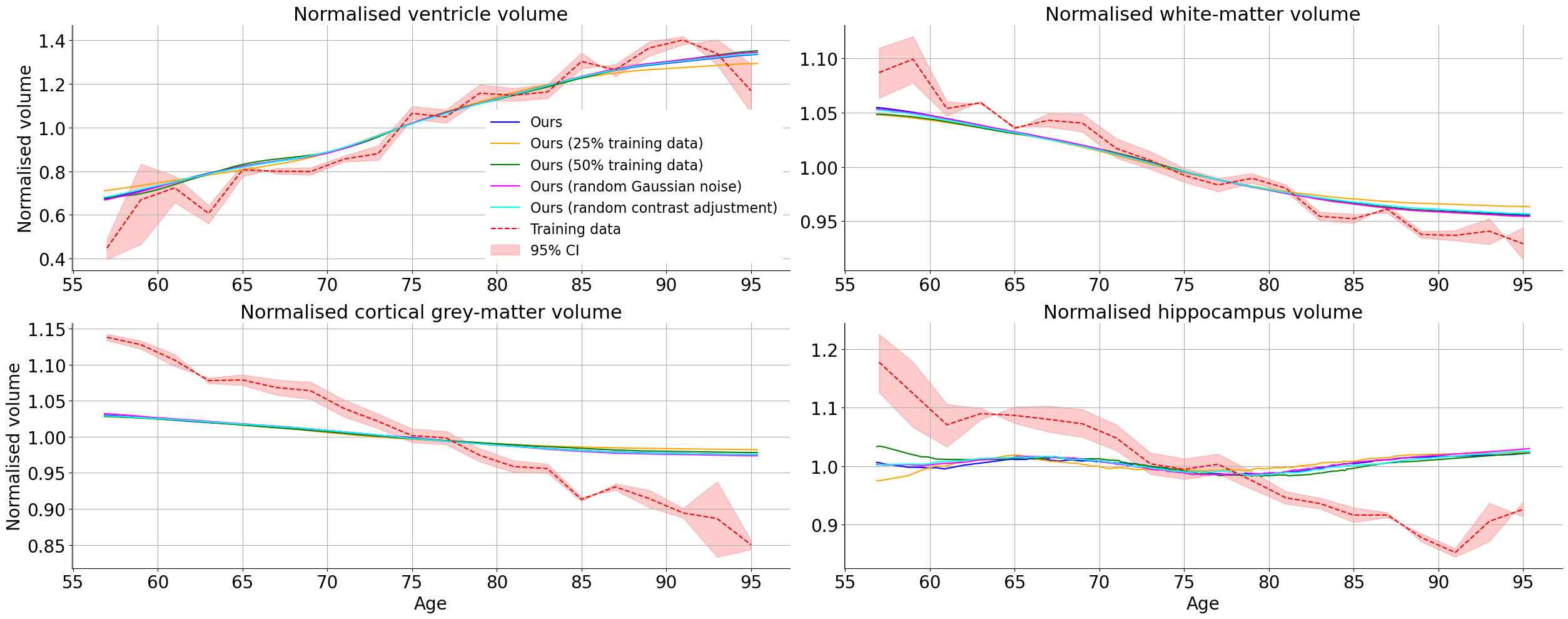

Supplement: Supplementary file 1 — Data S1. [file HBM-46-e70229-s002.zip › hbm70229-sup-0001-Supinfo/figure_3.png]

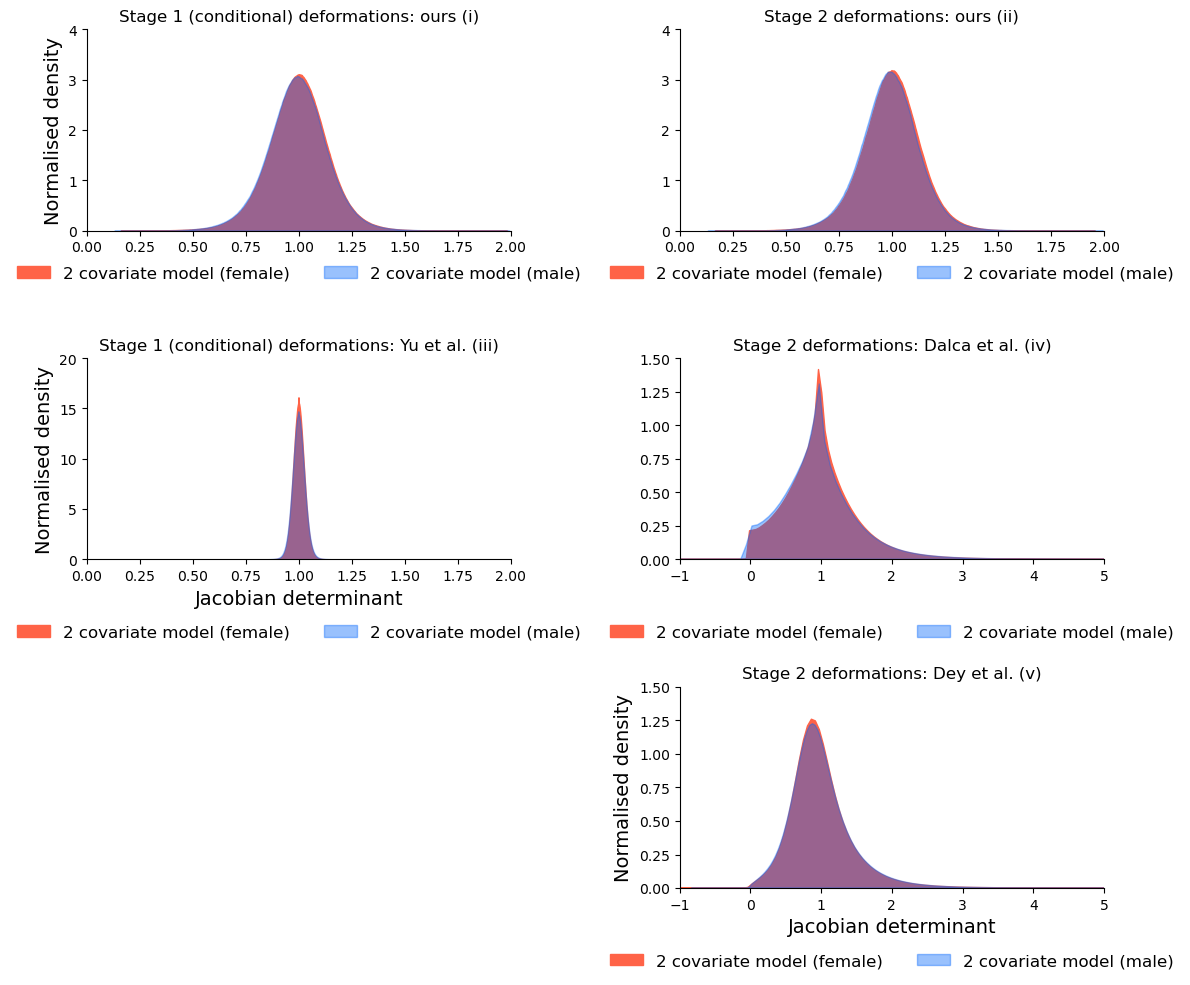

Supplement: Supplementary file 1 — Data S1. [file HBM-46-e70229-s002.zip › hbm70229-sup-0001-Supinfo/figure_4.png]

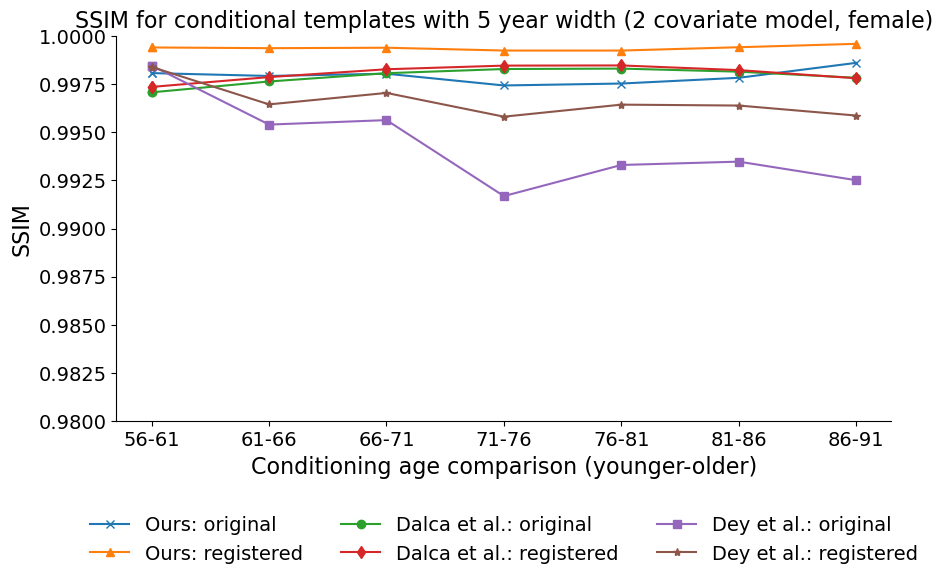

Supplement: Supplementary file 1 — Data S1. [file HBM-46-e70229-s002.zip › hbm70229-sup-0001-Supinfo/figure_5a.png]

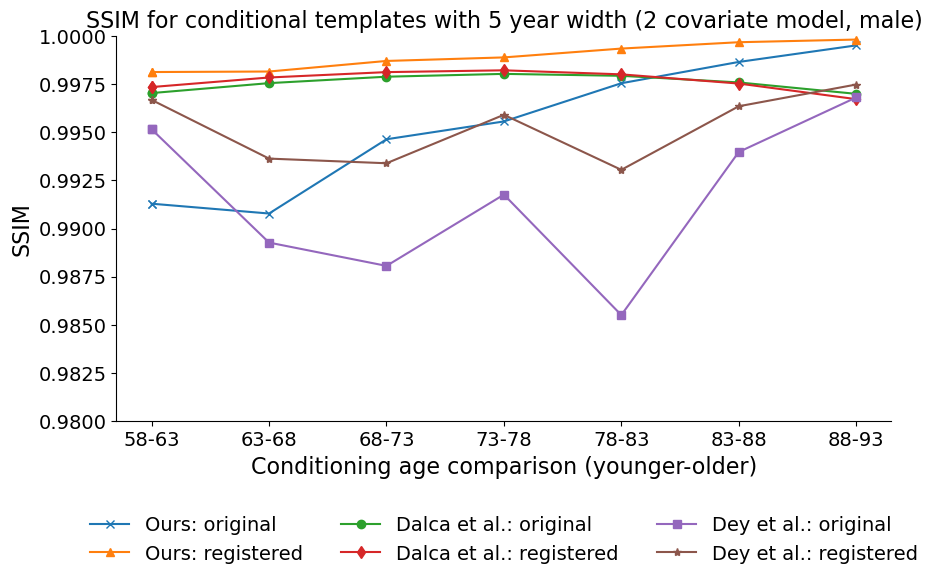

Supplement: Supplementary file 1 — Data S1. [file HBM-46-e70229-s002.zip › hbm70229-sup-0001-Supinfo/figure_5b.png]

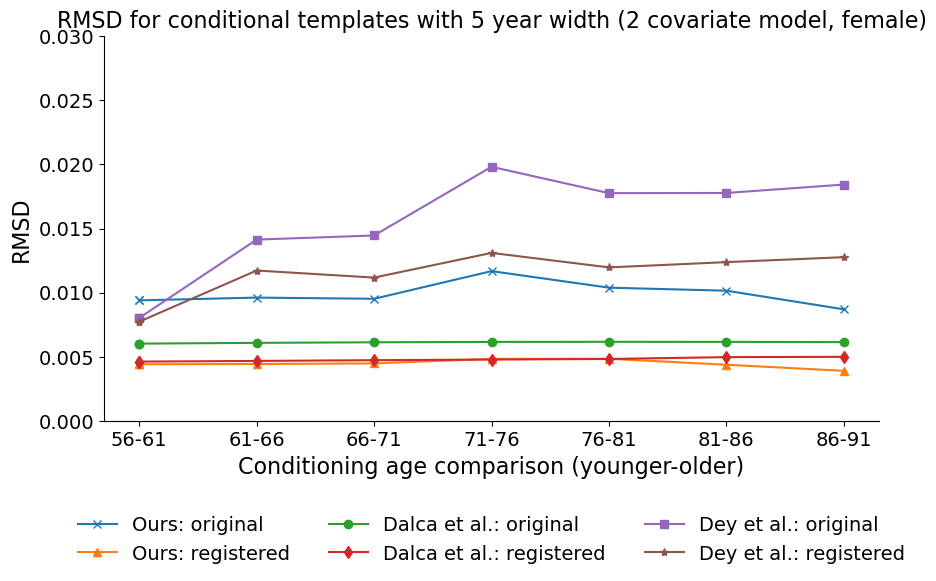

Supplement: Supplementary file 1 — Data S1. [file HBM-46-e70229-s002.zip › hbm70229-sup-0001-Supinfo/figure_5c.png]

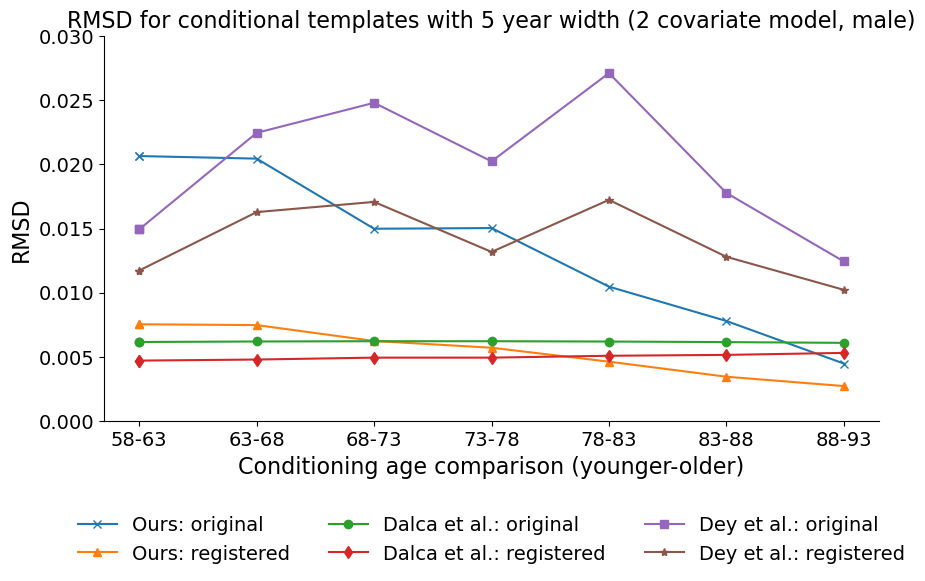

Supplement: Supplementary file 1 — Data S1. [file HBM-46-e70229-s002.zip › hbm70229-sup-0001-Supinfo/figure_5d.png]

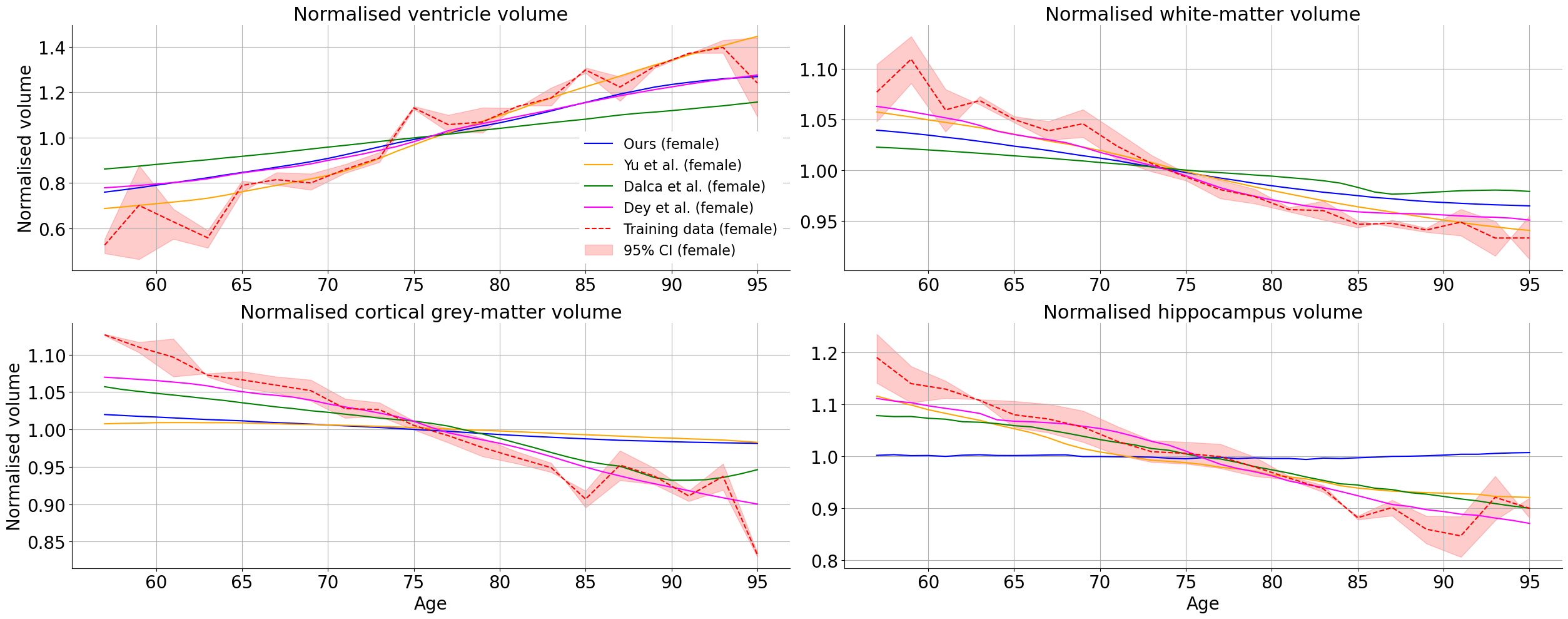

Supplement: Supplementary file 1 — Data S1. [file HBM-46-e70229-s002.zip › hbm70229-sup-0001-Supinfo/figure_6a.png]

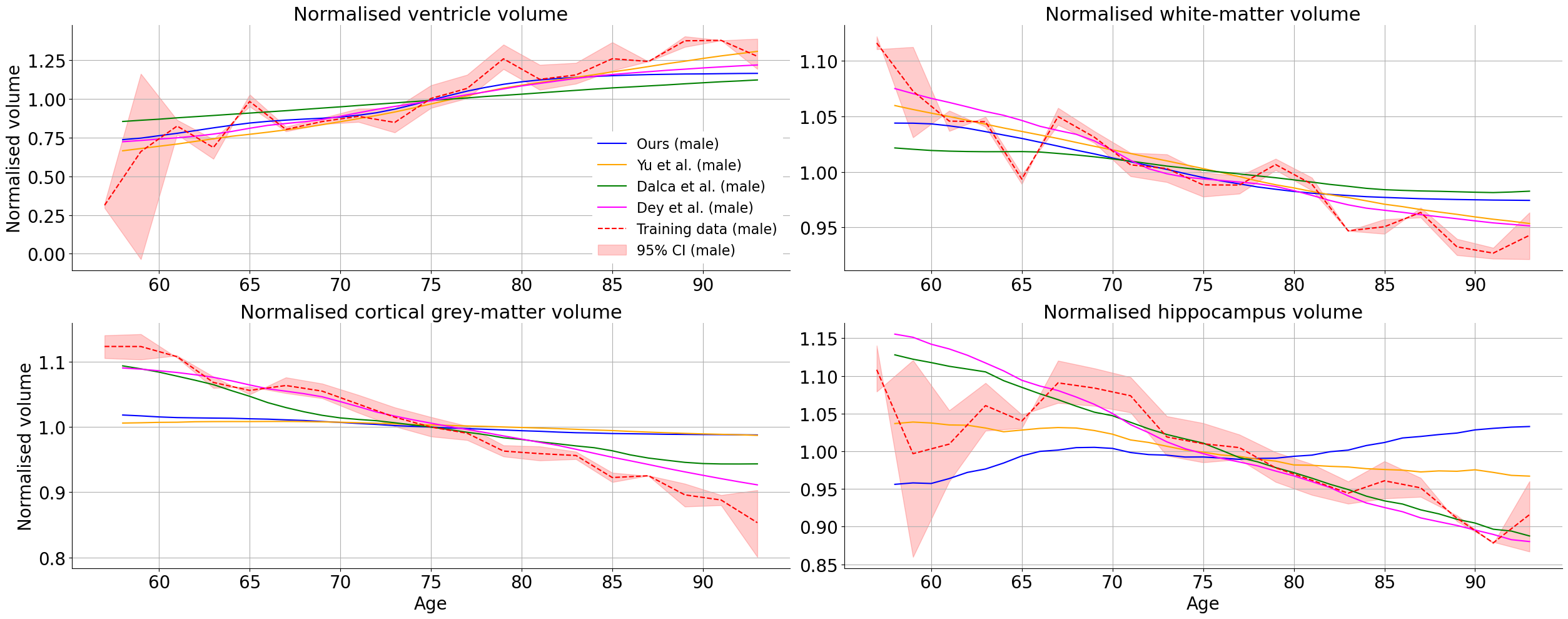

Supplement: Supplementary file 1 — Data S1. [file HBM-46-e70229-s002.zip › hbm70229-sup-0001-Supinfo/figure_6b.png]

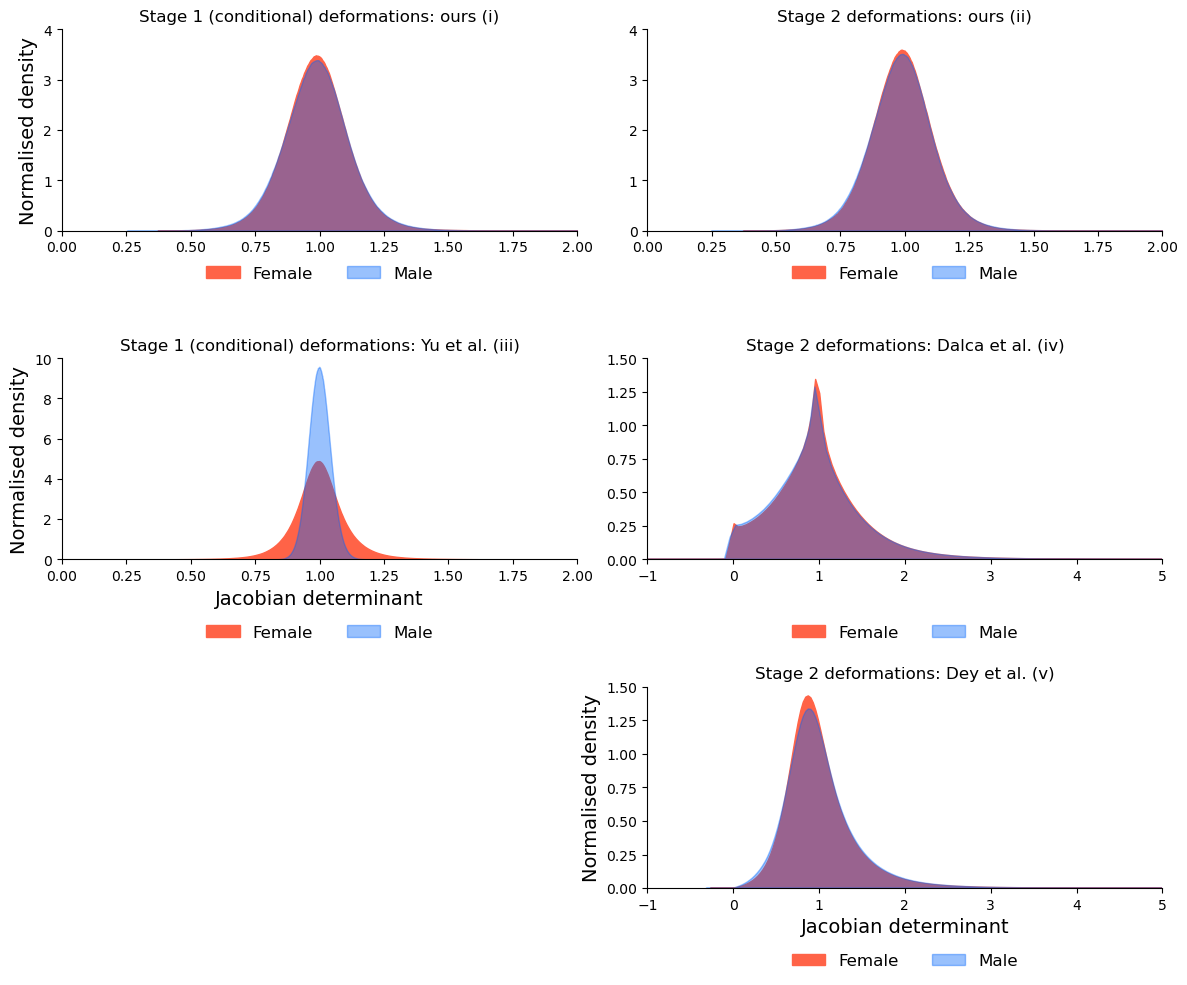

Supplement: Supplementary file 1 — Data S1. [file HBM-46-e70229-s002.zip › hbm70229-sup-0001-Supinfo/figure_7.png]

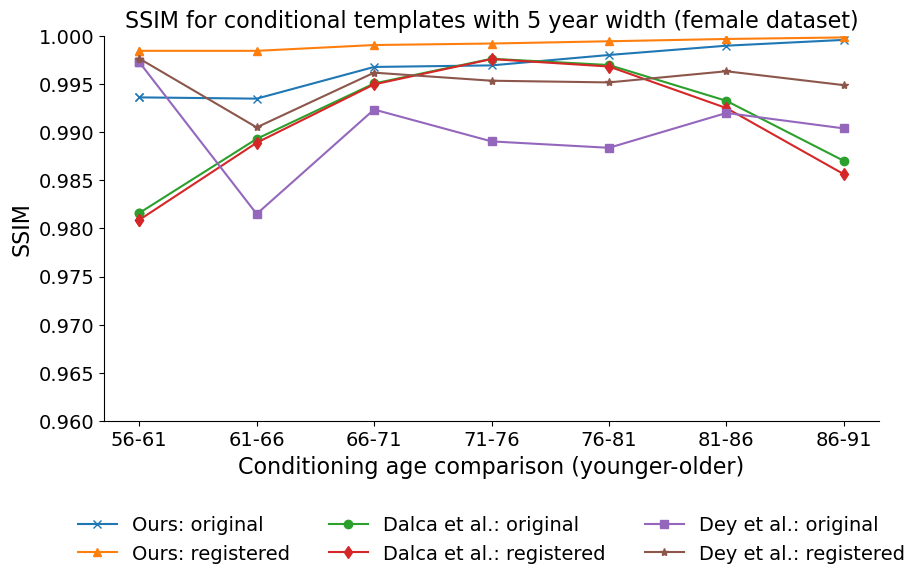

Supplement: Supplementary file 1 — Data S1. [file HBM-46-e70229-s002.zip › hbm70229-sup-0001-Supinfo/figure_8a.png]

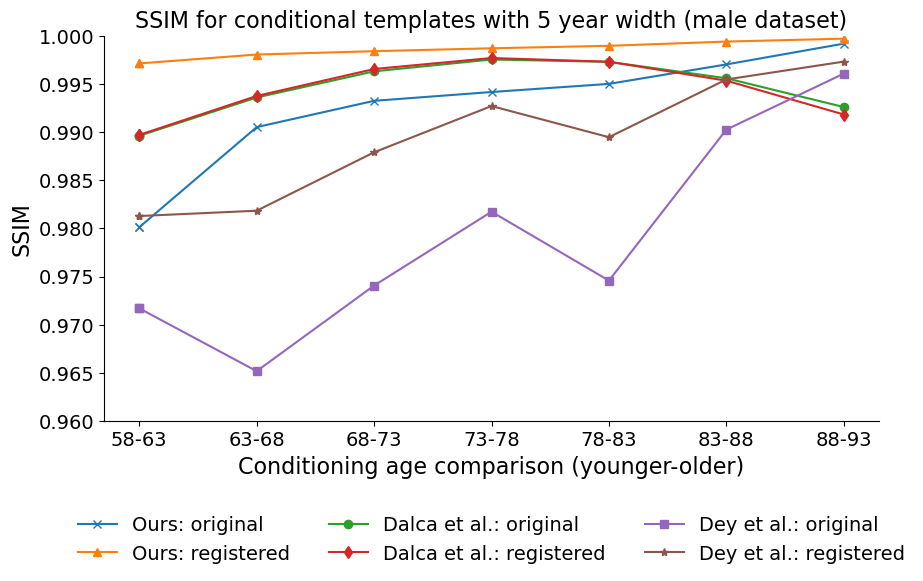

Supplement: Supplementary file 1 — Data S1. [file HBM-46-e70229-s002.zip › hbm70229-sup-0001-Supinfo/figure_8b.png]

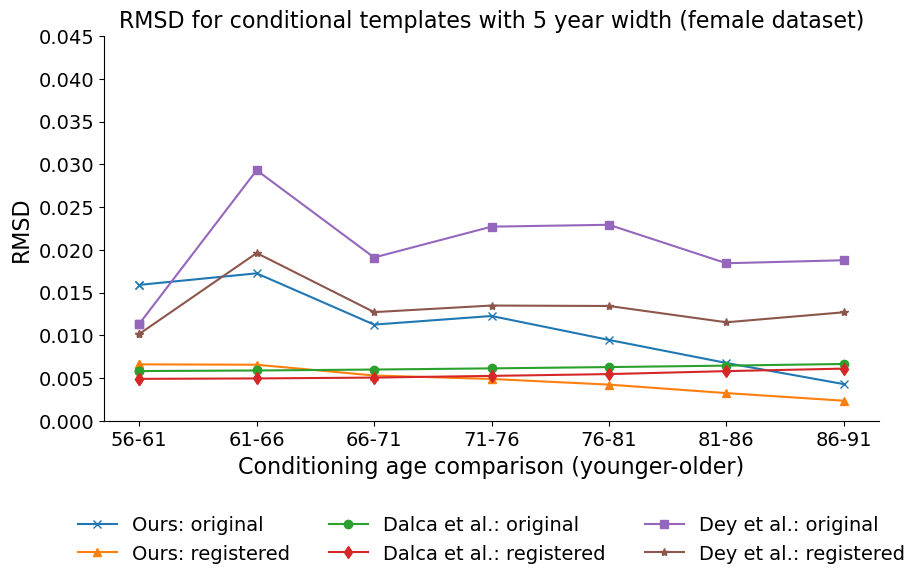

Supplement: Supplementary file 1 — Data S1. [file HBM-46-e70229-s002.zip › hbm70229-sup-0001-Supinfo/figure_8c.png]

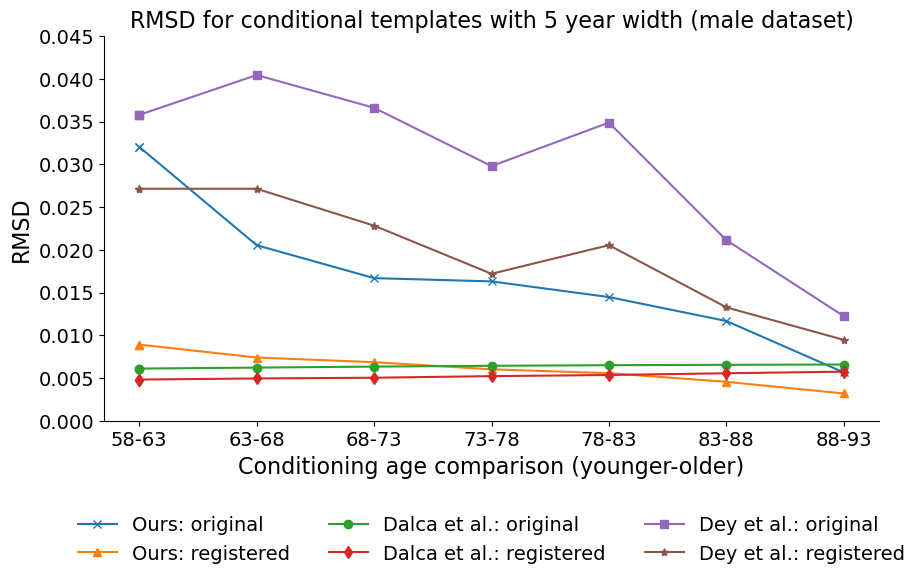

Supplement: Supplementary file 1 — Data S1. [file HBM-46-e70229-s002.zip › hbm70229-sup-0001-Supinfo/figure_8d.png]

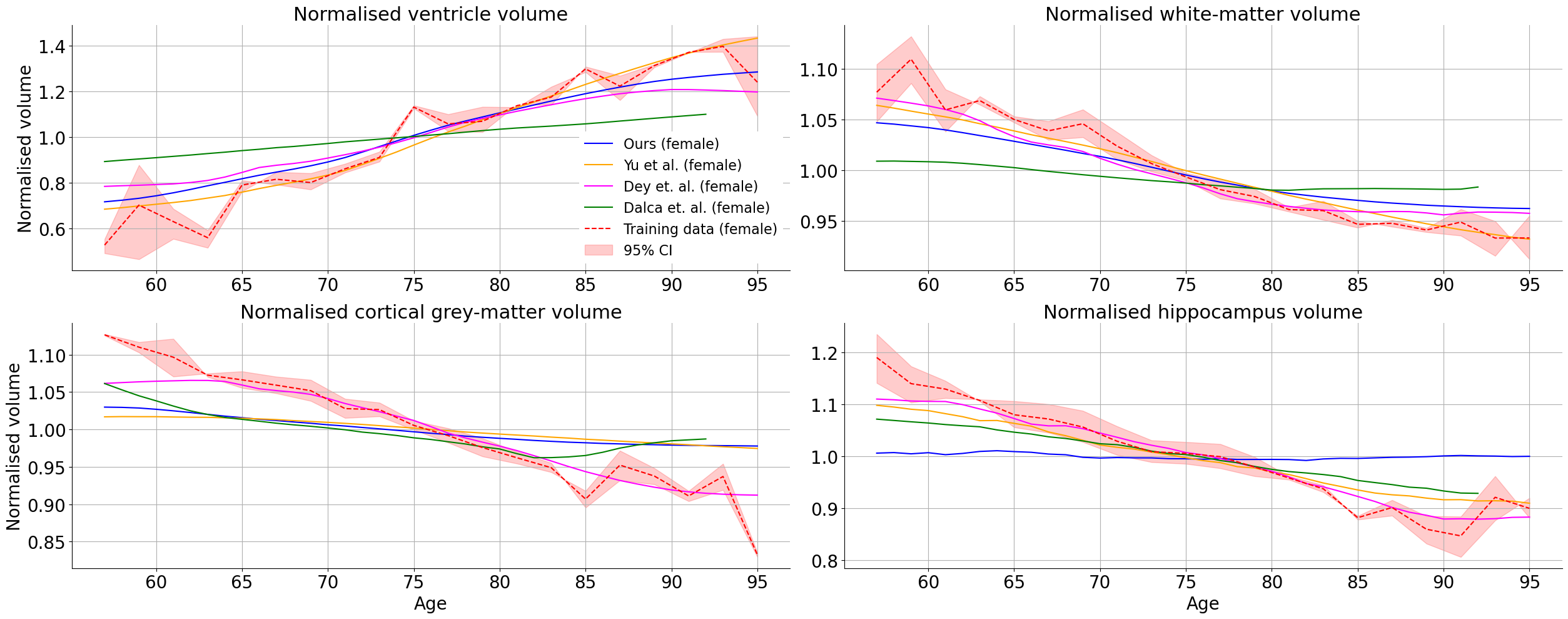

Supplement: Supplementary file 1 — Data S1. [file HBM-46-e70229-s002.zip › hbm70229-sup-0001-Supinfo/figure_9a.png]

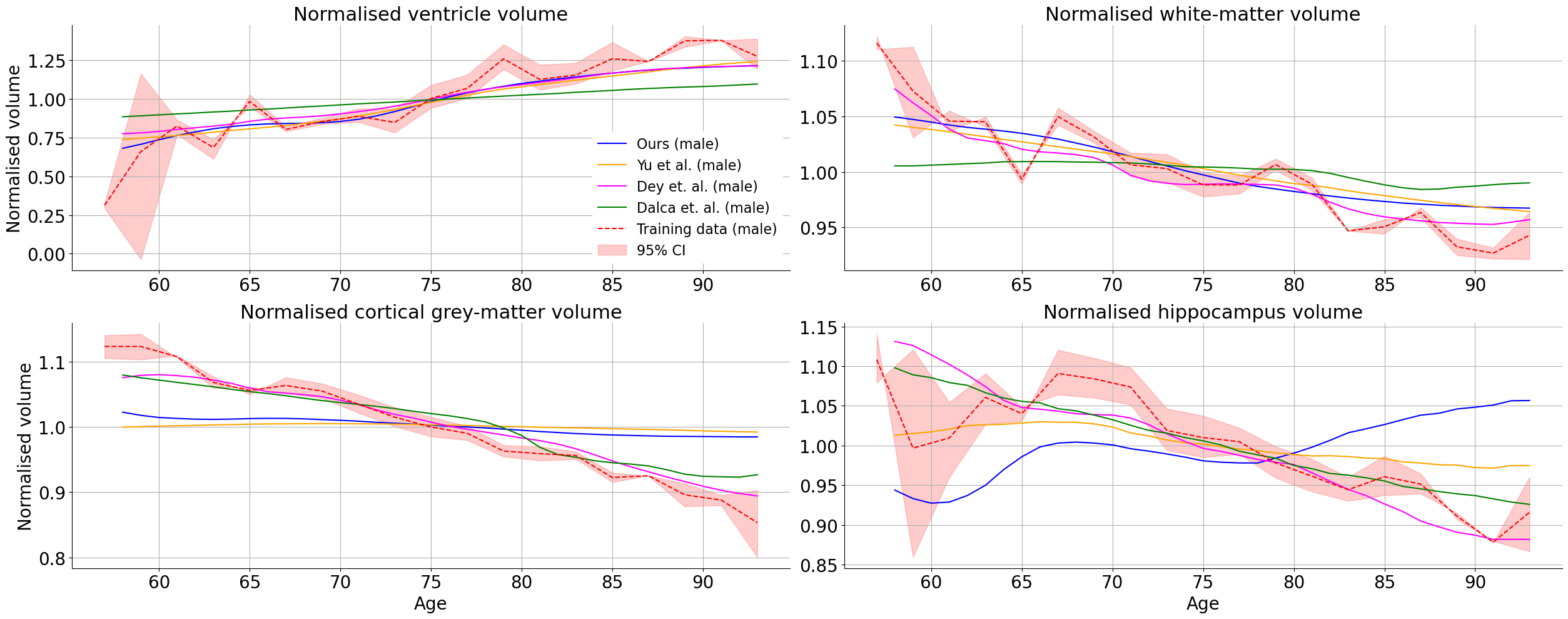

Supplement: Supplementary file 1 — Data S1. [file HBM-46-e70229-s002.zip › hbm70229-sup-0001-Supinfo/figure_9b.png]
